# An Unusual Case of Acute Carpal Tunnel Syndrome

**DOI:** 10.7759/cureus.20852

**Published:** 2021-12-31

**Authors:** Simranjit Singh, Fnu Sanna, Natasha Singh, Ramesh Adhikari, Vinod Kumar

**Affiliations:** 1 Department of Internal Medicine, Indiana University School of Medicine, Indianapolis, USA; 2 Hospital Medicine, Franciscan Health, Lafayette, USA; 3 Geriatrics, Brown University, Providence, USA

**Keywords:** direct acting oral anticoagulant, bleeding risk, hematoma, atraumatic, systemic anticoagulation, rivaroxaban, carpal tunnel syndome

## Abstract

Acute atraumatic carpal tunnel syndrome (CTS) that results from a hematoma as a complication of oral anticoagulation use is a highly uncommon presentation. CTS is a common type of peripheral compression neuropathy, with CTS's acute presentation being less common than chronic. The acute type is commonly caused either by recent trauma, including fractures of the distal radius and carpal dislocations, atraumatic etiologies like infections, or inflammatory conditions that increase the pressure in the carpal tunnel. Timely diagnosis of acute CTS is essential, as often surgical decompression is required if symptoms do not improve within hours.

A 79-year-old female presented to the ED with a past medical history significant for stroke, paroxysmal atrial fibrillation on rivaroxaban, and hypertension. She complained of a one-day history of left wrist pain, swelling, and restricted range of motion, associated with numbness in the median nerve distribution and weakening of the handgrip. The patient denied any trauma or unusual physical activity. CCT imaging of the left upper extremity showed soft tissue expansion around the flexor pollicis longus proximal to and just distal to the carpal tunnel consistent with dissecting hematoma within the flexor compartment. The orthopedics hand team evaluated the patient. Her rivaroxaban was held, and she was monitored for 24 hours in the hospital. The next day, she almost had a complete resolution of her symptoms. She was discharged home with a close follow-up.

There are various atraumatic causes of acute CTS. Spontaneous atraumatic hematoma occurring in the forearm's flexor compartment and resulting in acute CTS is extremely uncommon. In contrast to chronic CTS, acute CTS requires urgent carpal tunnel release to prevent irreversible median nerve damage. Anticoagulants in such cases increase the bleeding risk. This case highlights the importance of considering CTS into the differential diagnosis of someone on an anticoagulant and presenting with acute wrist swelling and pain. Despite the absence of any direct trauma, timely diagnosis of this condition is prudent and greatly affects the outcomes.

## Introduction

Development of hematoma as a complication of oral anticoagulation leading to carpal tunnel syndrome (CTS) is a rare condition. There are only a few case reports in the published literature. It is a rapidly progressive condition and often associated with severe pain and neurological deficits. The urgent carpal tunnel release might be needed to avoid irreversible neurological damage.

Acute CTS is an uncommon diagnosis, primarily related to blunt trauma. Unlike chronic CTS, immediate surgical decompression (carpal tunnel release) is required for acute CTS to avoid serious complications [1]. Acute CTS could also be related to hemorrhagic, vascular, rheumatological conditions, and bleeding disorders. A carpal tunnel is a close passageway surrounded by bones and ligaments, and symptoms quickly develop due to increased pressure in the space [2, 3]. Acute CTS has the characteristic pain due to median nerve compression and is associated with dysesthesia. Acute CTS symptoms progress over hours, whereas chronic CTS has a more insidious onset of symptoms.

Acute CTS can be managed with nonsurgical measures, such as elevation, dressing or cast release, compression wrapping, or observation for a few hours. However, in case of lack of improvement of symptoms within hours or a day or two at the most, surgical decompression to relieve carpal tunnel pressure is required [4, 5].

Very few case reports cite hematoma development due to intake of oral anticoagulants resulting in CTS [6,7]. However, it is often associated with severe pain and neurological deficits.

CTS itself is a common type of peripheral compression neuropathy. More commonly, CTS is a chronic disease with slow progression. CTS' acute presentation is significantly less common than chronic, mainly related to wrist trauma, fractures of the distal radius, and carpal bones dislocations. Rarely, acute CTS is caused by atraumatic etiologies such as inflammation, infections, or iatrogenic events that lead to increases in carpal tunnel pressure [8, 9, 10, 11].

## Case presentation

**Figure 1:**
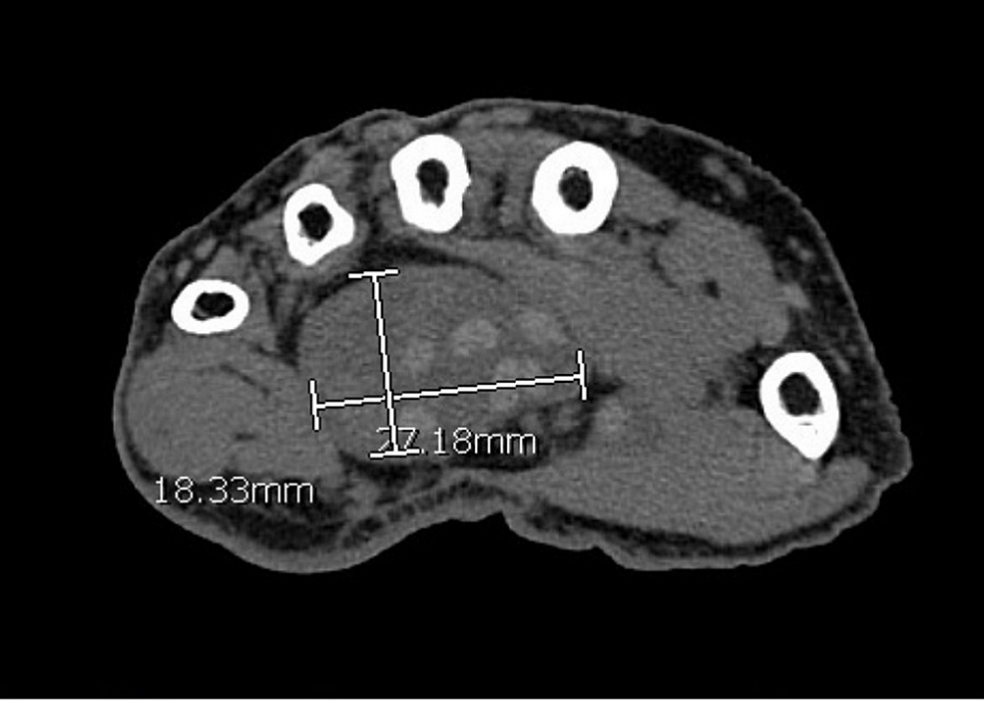
Expansion of the flexor compartment just distal to the carpal tunnel.

**Figure 2:**
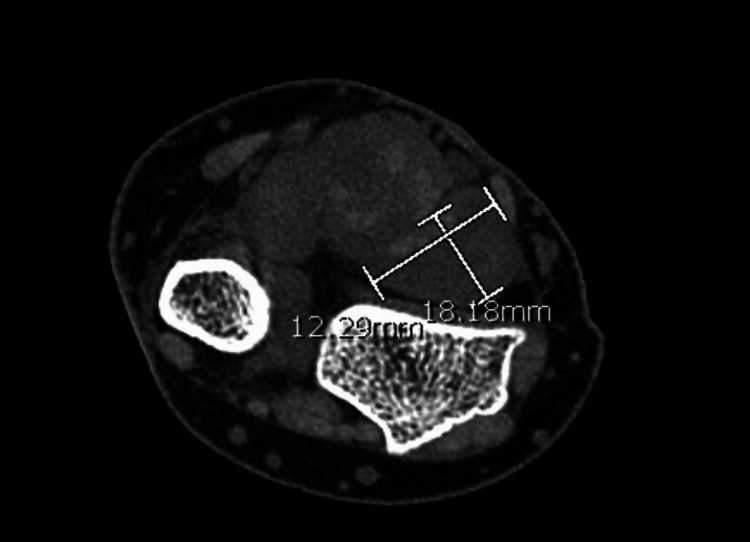
Soft tissue expansion around the flexor pollicis longus proximal to the carpal tunnel.

**Figure 3:**
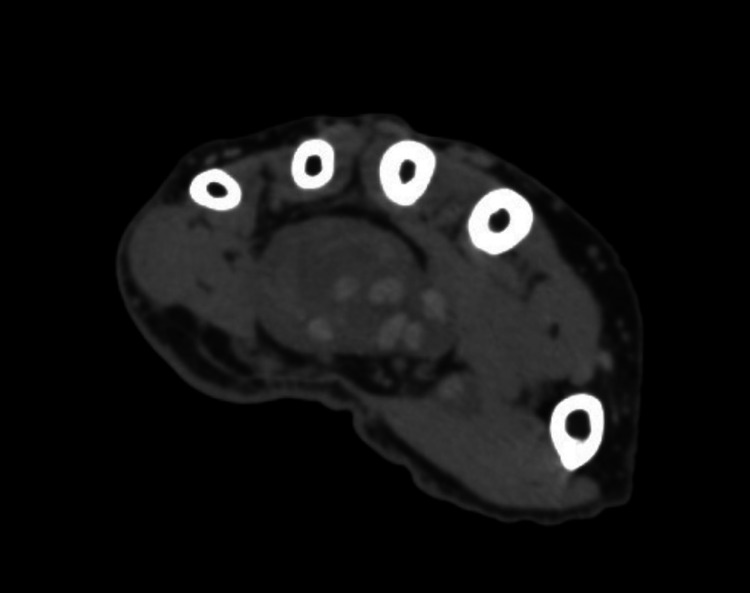
Carpal tunnel hematoma.

**Figure 4:**
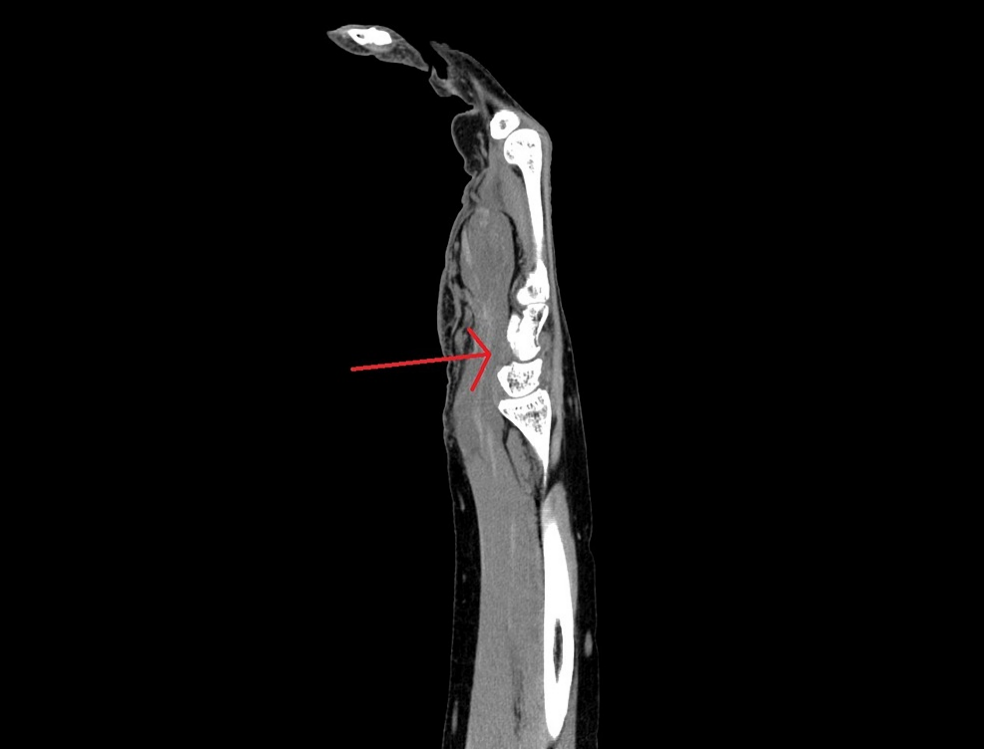
Longitudinal view of forearm showing the flexor compartment hematoma.

**Figure 5:**
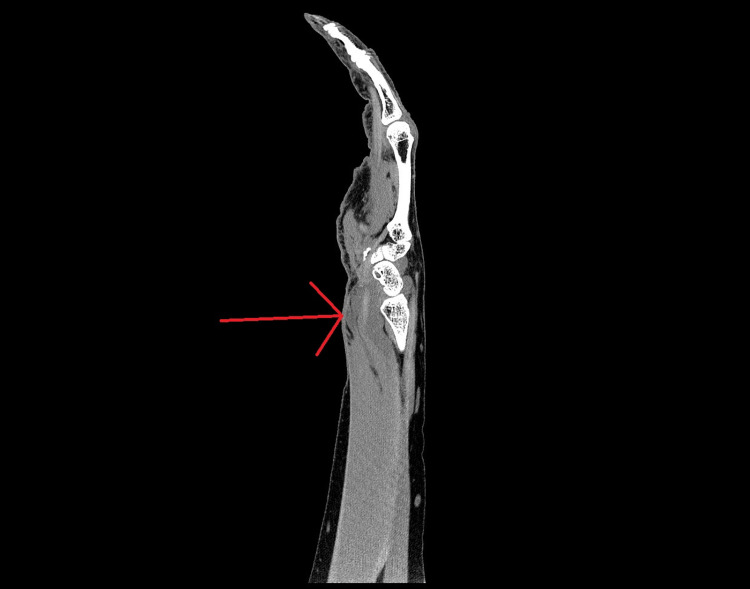
Flexor compartment hematoma involving the carpal tunnel.

A 79-year-old female, with a past medical history of stroke, hypertension, paroxysmal atrial fibrillation on rivaroxaban, obstructive sleep apnea, and asthma, presented to the ED. She had a one-day history of left wrist pain, swelling, and a restricted range of motion. Wrist pain was acute in onset and severe in intensity. Since its onset, she had worsening swelling and weakness of left-hand muscles. In addition, she reported tingling and numbness in the fingers of the median nerve distribution. The patient denied any trauma or unusual physical activity preceding the onset of symptoms. The pain had no alleviating factors other than over-the-counter acetaminophen and ibuprofen pain medicines. Symptoms were exacerbated by movement, especially wrist flexion. Her digits were resting in a clawing position. The patient had no fever, chills, respiratory, cardiovascular, GI, or genitourinary symptoms. Her vitals were within the normal range. Her complete blood count, comprehensive metabolic profile, INR, TSH, and C-reactive protein were within the normal range. CT imaging of the left upper extremity showed soft tissue expansion around the flexor pollicis longus proximal to and just distal to the carpal tunnel consistent with dissecting hematoma within the flexor compartment (Figures 1-5). The orthopedics hand team evaluated the patient.

She was diagnosed with acute CTS from anticoagulant-related hematoma based on clinical and radiographic findings. No other cause was identified. The timing of symptom progression, radiographic findings, and absence of any history of inflammatory or autoimmune condition ruled out the possibility of inflammatory etiology of CTS. In addition, the absence of trauma history and unremarkable skeletal survey on radiographic imaging ruled out traumatic causes of acute CTS.

She had improvement of her symptoms with oral oxycodone and IV hydromorphone pain medicines, limb elevation, compression wrapping, and rest. It was decided to follow conservative nonoperative management with close monitoring. Her rivaroxaban was held, and she was monitored for 24 hours in the hospital. The next day, the patient had almost complete resolution of her symptoms. Her paresthesia and pain symptoms were resolved, and she had improved range of motion. She was discharged home with a close follow-up. Rivaroxaban was not restarted at the time of discharge.

## Discussion

Acute atraumatic CTS is a rare condition. In the presented case, the patient has been on rivaroxaban. The patient presented with acute onset arm swelling and severe pain. As a complication from anticoagulation, the hematoma created local pressure resulting in acute CTS by direct pressure on the median nerve [2].

Acute CTS in association with rivaroxaban use is extremely rare. The patient had a clear diagnosis of acute CTS based on the characteristic findings of severe pain, rapid onset of symptoms, median nerve distribution of symptoms, and absence of other risk factors. The patient was considered to have sustained relatively minor vascular trauma in the synovial flexor sheath. Subsequently, the median nerve got compressed due to rapidly expanding hematoma. The diagnosis was clear based on the history, physical exam, and radiological findings. Therefore, measuring carpal tunnel compartment pressures was not necessary. The patient was treated nonsurgically with the resolution of symptoms. If the patient had no rapid improvement (within hours or a day or two at the most) of symptoms, the orthopedics team was on standby for an emergent surgical decompression. Delay in surgical decompression can result in prolonged or incomplete recovery in acute CTS cases [5].

Rivaroxaban is an oral, direct thrombin inhibitor with a half-life of 5-9 hours. Unlike warfarin, it does not require regular laboratory monitoring. New oral anticoagulants like rivaroxaban are considered to have fewer adverse side effects than vitamin K antagonists. However, spontaneous hemorrhages have been reported [12,13].

The few cases in the published literature regarding atraumatic CTS in patients on rivaroxaban had certain underlying conditions which potentially acted as predisposing risk factors for the hemorrhage. Komura S et al. described a case of distal radioulnar joint arthritis with dorsal dislocation and spur formation of the ulnar head resulting in flexor tendon abrasion. The patient was on apixaban and presented with hematoma in the carpal tunnel, resulting in recurrent acute CTS [11]. In the case described by Weschenfelder W et al., the patient had longstanding and progressive movement restrictions of the finger flexion, beginning at the thumb. Authors suspected tendon erosion or rupture as a cause of the hematoma. The intraoperative findings showed erosion of the long flexor tendon of the index finger along with signs of local bleeding, further demonstrating evidence of a recent event [14]. Interestingly, the presented case of atraumatic acute CTS from rivaroxaban use was spontaneous without any identified predisposing condition.

It is recommended that acute CTS requires prompt carpal tunnel release as delay can lead to irreversible median nerve damage and poor outcomes [15]. Our patient had acute CTS secondary to carpal tunnel hematoma. She showed improvement of symptoms upon arrival to the hospital. Therefore, surgical intervention was not needed, and instead, she was closely monitored in the hospital with conservative management, including limb elevation, pain medicines, and compression wrappings. She had a spontaneous resolution of the symptoms. She was being treated with rivaroxaban for her paroxysmal atrial fibrillation. Rivaroxaban has been reported effective for preventing systemic embolism, cerebral infarction, and prevention of stroke. However, some patients have experienced spontaneous hematoma formation. Although the presented patient was not on any antiplatelet drugs, the use of antiplatelet along with anticoagulants has shown a further increased risk of spontaneous hematoma. Therefore, the patient was discharged from the hospital without restarting rivaroxaban. There are no clearly established guidelines about whether and when anticoagulation can be restarted. The decision to restart anticoagulation is on a case-by-case basis and depends on the risk of rebleed and risk of thrombotic events.

## Conclusions

Spontaneous atraumatic hematoma occurring in the flexor compartment of the forearm resulting in acute CTS is an uncommon condition. Compared to chronic CTS, acute CTS needs urgent carpal tunnel release to prevent irreversible median nerve damage. Conservative therapy of monitoring, controlling symptoms, limb elevation, and stopping anticoagulation could be considered depending on the severity of symptoms and clinical progression. Nevertheless, authors recommend a low threshold for surgical decompression of carpal tunnel urgently, in case conservative management does not improve the symptoms. Anticoagulant therapy modification postoperatively or post-hospital discharge needs to be considered on a case-by-case basis.

In conclusion, atraumatic acute CTS is a rare type of median nerve disorder. Anticoagulants increase the bleeding risk, including the occurrence of spontaneous hematoma formation. This case highlights the importance of considering CTS into the differential diagnosis when someone presents with acute wrist swelling and pain, despite the absence of any direct trauma. The timely diagnosis and intervention are crucial in improving the outcomes. Delaying treatment can lead to permanent median nerve damage. The use of anticoagulants with or without antiplatelet should further raise the suspicion of spontaneous hematoma, especially in the absence of traumatic etiology and acute onset of symptoms. There needs to be a low threshold for surgical intervention, including the release of carpal tunnel pressure. In the presented case, the patient had resolution of symptoms with conservative (nonsurgical) management. The presented case is the first reported case of atraumatic acute CTS with rivaroxaban use.
